# Supplementary material for: Hydrogen Peroxide Increases during Endodormancy and Decreases during Budbreak in Grapevine (Vitis vinifera L.) Buds
Source: Antioxidants (Basel). 2021 May 29;10(6):873. doi: 10.3390/antiox10060873 (PMC8228137; doi:10.3390/antiox10060873)
Supplement: Supplementary file 1 [file antioxidants-10-00873-s001.zip › antioxidants-1218153-supplementary.pdf]

Table S1. *V. vinifera* peroxidase genes (*VvPODs*) whose expression is regulated by ABA.

Peroxidase Genes	References
VvPOD2	[43, 44]
VvPOD4	[43]
VvPOD14	[44]
VvPOD18	[44]
VvPOD19	[44]
VvPOD20	[44]
VvPOD21	[44]
VvPOD22	[44]
VvPOD23	[43,44]
VvPOD24	[43,44]
VvPOD30	[43]
VvPOD31	[44]
VvPOD32	[43,44]
VvPOD36	[43,44]
VvPOD37	[44]
VvPOD38	[43, 44]
VvPOD40	[44]
VvPOD46	[44]
